# Mechanistic Study of Synergistic Antimicrobial Effects between Poly (3-hydroxybutyrate) Oligomer and Polyethylene Glycol

**DOI:** 10.3390/polym12112735

**Published:** 2020-11-18

**Authors:** Ziheng Zhang, Jun Li, Linlin Ma, Xingxing Yang, Bin Fei, Polly H. M. Leung, Xiaoming Tao

**Affiliations:** 1 Research Center for Smart Wearable Technology, Institute of Textiles and Clothing, The Hong Kong Polytechnic University, Hong Kong 999077, China; ziheng.zhang@connect.polyu.hk (Z.Z.); j.li@connect.polyu.hk (J.L.); linma@polyu.edu.hk (L.M.); u3004941@connect.hku.hk (X.Y.); bin.fei@polyu.edu.hk (B.F.); 2 Department of Health Technology and Informatics, Hong Kong Polytechnic University, Hong Kong 999077, China; polly.hm.leung@polyu.edu.hk

**Keywords:** poly (3-hydroxybutyric acid), oligomer, polyethylene glycol, antimicrobial agent, synergistic antimicrobial effect

## Abstract

Extended from our previous finding that poly (3-hydroxybutyrate) (PHB) oligomer is an effective antimicrobial agent against gram-positive bacteria, gram-negative bacteria, fungi and multi-drug resistant bacteria, this work investigates the effect of polyethylene glycol (PEG) on the antimicrobial effect of PHB oligomer. To investigate and explain this promoting phenomenon, three hypothetic mechanisms were proposed, that is, generation of new antimicrobial components, degradation of PHB macromolecules and dissolution/dispersion of PHB oligomer by PEG. With a series of systematic experiments and characterizations of high-performance liquid chromatography–mass spectrometry (HPLC-MS), it was deducted that PEG promotes the antimicrobial effect of PHB oligomer synergistically through dissolution/dispersion, owing to its amphipathy, which improves the hydrophilicity of PHB oligomer.

## 1. Introduction

Pathogens, e.g., bacteria, fungi and viruses, can cause serious diseases due to their high reproductivity and adaptability [1]. Antibiotics have been commonly used to prevent bacterial infection, whereas the overuse of them leads to antibiotic resistance, which has become a worldwide threat to human health. About 700,000 people die each year due to infections caused by multi-drug-resistant (MDR) bacteria [2,3]. Inorganic nanoparticles, e.g., nano-silver particles [2], have been verified to be highly effective against MDR bacteria; nevertheless, this type of antimicrobial agent potentially threatens human health and the environment [4]. Many new antibiotics and antimicrobial peptides have been developed for dealing with continuously-emerging MDR bacteria, but the development rate of novel antimicrobial agents cannot catch up with the enhancement of antibiotic resistance of MDR bacteria through bacterial surface modification, protease secretion and expression of efflux pumps [5,6]. It has been found that many essential oils (Eos) are effective at killing MDR bacteria and can be applied with antibiotics for enhance the efficacy synergistically [7,8]. However, the natural components of EOs cannot be controlled accurately, while their low durability and high volatility restrict their applications. 

We have reported previously that the synthesized poly (3-hydroxybutyrate) (S-PHB) oligomer, made by open-ring polymerization of beta-butyrolactone, possessed an excellent antimicrobial effect against gram-positive bacteria, gram-negative bacteria and fungi with a high reduction rate over 99.99%, as well as multi-drug resistant (MDR) bacteria (methicillin-resistant *S. aureus*, ATCC 43300) with reduction rate of 99.97% [9]. The antimicrobial mechanism consisted of the destruction of biofilm/cell walls/membranes, leakage of the intracellular content, change in the transmembrane potential and inhibition of protein activity, owing to its specific hydrophobicity and polarity. The PHB oligomer is degradable, durable, eco-friendly and safe, thus has significant advantages for the applications in healthcare field. Nevertheless, the material cost of synthesizing PHB (i.e., beta-butyrolactone) is relatively high compared with the fermented PHB powder from starch or sugar, in which the PHB oligomer can be extracted. The extracted PHB (E-PHB) oligomer exhibits a reduction rate of over 90% against gram-positive bacteria, gram-negative bacteria and fungi, which is insufficient to be used as high-effectiveness antimicrobial material. Therefore, it is important to find alternative ways to promote the effectiveness of E-PHB, in order to achieve both high performance and low cost. Two alternative methods can be explored, that is, the purification of an effective antimicrobial component [9,10,11,12] and the addition of a synergistic agent [13,14,15,16] with low cost.

In this work, we firstly attempted to promote the antimicrobial property of E-PHB by purification, and found surprisingly that purification reduced the antimicrobial effects of E-PHB rather than increased it as expected. We noticed that the elimination of polyethylene glycol (PEG) from the PHB powder extract might be the main reason, and that a synergistic antimicrobial effect between the PHB oligomer and PEG might exist and should be revealed. 

We then proposed three hypothetic mechanisms, including the generation of new antimicrobial components, the degradation of PHB macromolecules and the dissolution/dispersion of PHB oligomer by PEG during the process of purification. The experimental results show that the hypothesis of dissolution/dispersion of PHB oligomer by PEG dominates the synergistic antimicrobial effect. This work demonstrates a way to enhance antimicrobial effect of PHB oligomer and other antimicrobial agents with low-cost amphipathic material (e.g., PEG) through improving hydrophilicity.

## 2. Materials and Methods

### 2.1. Materials

Poly (3-hydroxybutyrate) (PHB) powder was provided by TianAn Biologic Materials Co., Ltd. in Ningbo, Zhejiang, China. Beta-butyrolactone (95.0%, TCI, Shanghai, China), aluminium isopropoxide (98.0%, TCI, Shanghai, China) and pyridine (99.0%, Acros Organics, NJ, USA) were used for chemically synthesizing PHB oligomer. PEG (M_w_ = 600, Acros Organics, NJ, USA) is applied for synergistic antimicrobial effect with PHB oligomer. The solvents, including chloroform, ethanol, methanol, dichloromethane (DCM) and n-hexane, were supplied by Anaqua (Hong Kong).

### 2.2. Preparation of PHB Oligomer by Chemical Synthesis

Beta-butyrolactone (0.86 g, 10 mmoL) was added to a mixed solution of pyridine (2 mL) and aluminum isopropoxide (0.2 g, 1 mmoL), then stirred at 65 °C under nitrogen atmosphere for 48 h [17]. HCl solution (2 m, 20 mL) was injected to quench the reaction, prior to an extraction with DCM. PHB oligomer was obtained after removal of the DCM by vacuum evaporation and separation by column chromatography (DCM/n-hexane = 5:1, v:v) [18]. 

### 2.3. Preparation of PHB Oligomer by Extraction (E-PHB) and Compounding with PEG

Mixture of PHB powder (10 g) and chloroform (200 mL) was refluxed for 12 h, then 1 L of methanol was added into the viscous solution, followed by filtration for removing the PHB polymer with high molecular weight (collected as PHB bulk). Afterwards, the raw PHB oligomer was obtained after the removal of solvents through rotary evaporation and further purified by column chromatography (DCM/n-hexane of 1:1 to methanol/DCM of 1:2). For compounding, the E-PHB was mixed with PEG (1:1) and stirred under 150 °C for 4 h.

### 2.4. Interaction of PHB Powder and PEG

Bio-based PHB powder was mixed with PEG (1:1) and stirred under 150 °C for 4 h. Afterwards, ethanol was added to dissolve the mixture and residual PHB (solid) was removed by centrifugation. After removing the solvent, yellow oil was obtained as a mixture for synergistic antimicrobial test.

### 2.5. Notions of Materials, Products and Experiments

**Figure 1:**
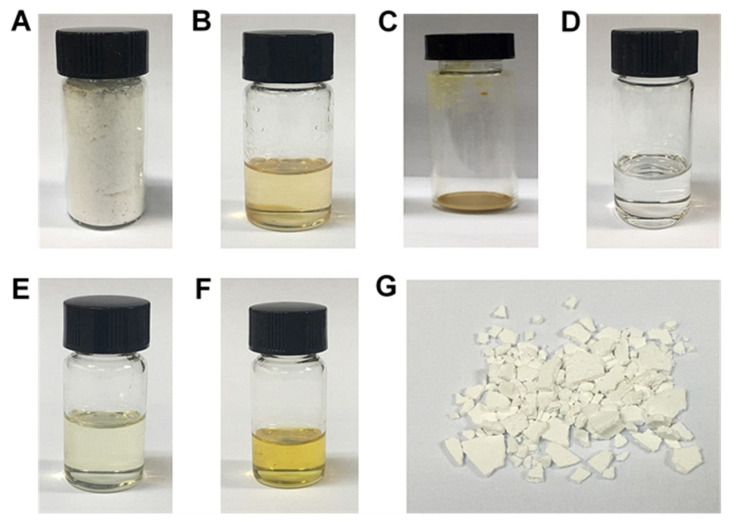
Images of poly (3-hydroxybutyrate) (PHB) and polyethylene glycol (PEG). (**A**) PHB powder. (**B**) synthesized poly (3-hydroxybutyrate) (S-PHB). (**C**) Extracted PHB (E-PHB) or extracted and purified PHB (EP-PHB). (**D**) PEG. (**E**) Liquid product of reaction of PHB powder and PEG under room temperature. (**F**) Liquid product of reaction of PHB powder and PEG under 150 °C. (**G**) PHB bulk.

The materials, products and experiments mentioned in this work are summarized in Table 1 and Figure 1 for clarification. 

### 2.6. Characterization of PHB and PEG

HPLC-MS tests were carried out by Thermo Fisher Orbitrap Fusion Lumos Mass Spectrometer (Waltham, MA, USA). Solvent of methanol/dichloromethane (1:1, v:v) was adopted as the eluent.

### 2.7. Antimicrobial Activity Tests

The antimicrobial property of the oligomer against *Staphylococcus aureus* (*S. aureus*) ATCC No. 6538, *Klebsiella pneumoniae* (*K. pneumoniae*) ATCC No. 4352, and *Candida albicans* (*C. albicans*) (Beisi, Shanghai, China) ATCC No. 10231 was tested according to the shake flask method [19] with concentration of 10 mg/mL [18].

The microorganisms were cultured for 24 h at 37 °C and diluted to 10^5^ CFU mL^−1^ approximately with PBS. Next, 70 mL PBS solution and 0.75 g/1.5 g of PHB oligomer were added to each flask and 5 mL of working dilution of bacterial was incubated at each flask and cultured for 18 h. The bacteria incubated without PHB served as the control group. After incubation, the bacteria solution containing untreated control or treated group were diluted by gradient and plated on nutrient agar, culturing for 18 h at 37 °C. The antibacterial rate was calculated by the following equation:

Antibacterial rate = (1 − CFU_PHB_/CFU_control_) × 100% (1)

## 3. Results and Discussion

### 3.1. Discovery of Synergy between PHB Oligomer and PEG

For producing antimicrobial PHB oligomer with raw materials of lower cost, the extraction of PHB oligomer from PHB powder is preferable compared with chemical synthesis of PHB oligomer. However, the extracted PHB (E-PHB) oligomer possesses relatively lower antimicrobial reduction against *K. pneumoniae* and *C. albicans* than the synthesized PHB (S-PHB) oligomer, i.e., 94.26% and 91.95%, respectively (Table 1). As the PHB oligomer has been proved to be an effective antimicrobial agent, it is supposed that E-PHB oligomer with higher purity might achieve better antimicrobial effect; thus, the E-PHB oligomer is further purified by column chromatography to obtain extracted and purified PHB (EP-PHB) oligomer. Nevertheless, the EP-PHB oligomer with higher purity does not possess better antimicrobial effect than expected. Contrarily, it achieves only antibacterial reduction of 49.6% against *K. pneumoniae* and has no effect against *C. albicans*. 

**Figure 2:**
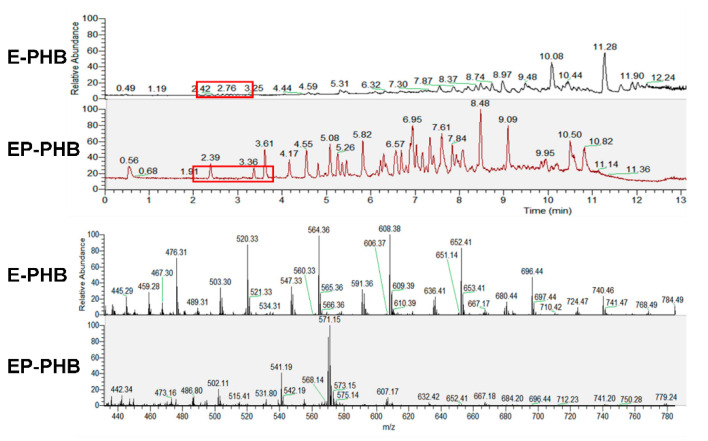
HPLC-MS spectrum of E-PHB and EP-PHB oligomer.

For investigating the reason why higher purity leads to low antimicrobial effect, HPLC-MS tests are tested for both E-PHB oligomer and EP-PHB oligomer (Figure 2). It can be seen that a polymer component with repeating unit of 44 exists in E-PHB rather than EP-PHB, which is deduced as polyethylene glycol (PEG), as it has been found previously that only C, H and O elements exist in the sample [18]. Additionally, this component appears early (2~3.5 min) in the HPLC spectra, which means it has relatively high polarity, which further confirms the deduction.

**Figure 3:**
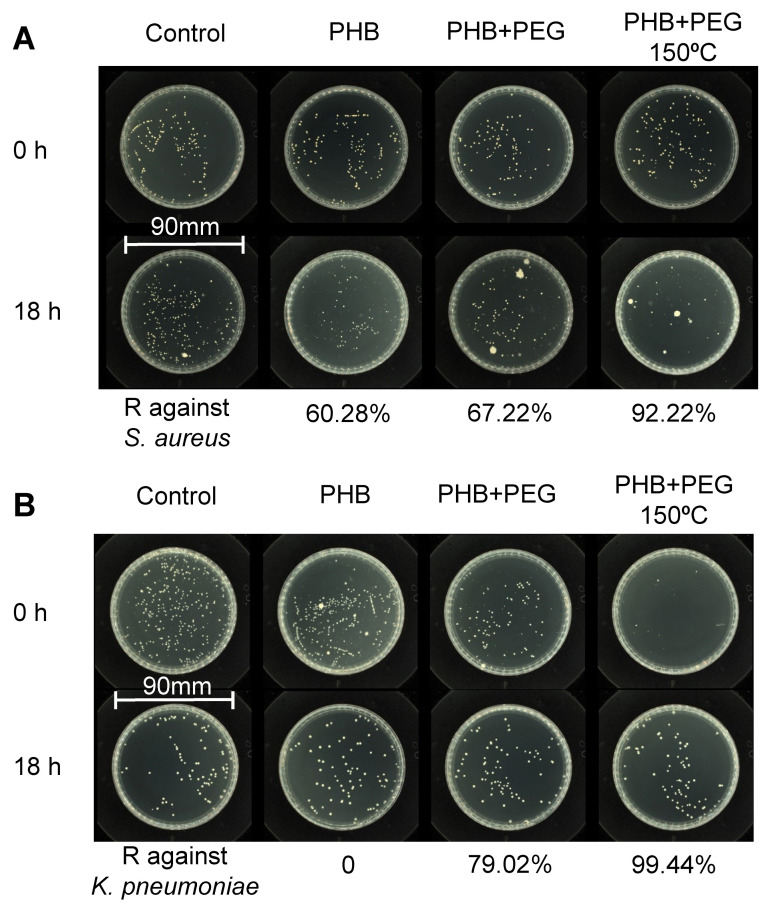
Antimicrobial test for PHB powder, mixture of PHB powder and PEG reacted under room temperature, and mixture of PHB powder and PEG reacted under 150 °C. (**A**) Antimicrobial test against *S. aureus*. (**B**) Antimicrobial test against *K. pneumoniae*.

Therefore, it is hypothesized that the existence of PEG in PHB oligomer can promote the antimicrobial effect of PHB, whereas the elimination of PEG after purifying E-PHB decreases its antimicrobial performance. A verifying experiment was carried out to mix EP-PHB oligomer and PEG under 150 °C for 4 h and test the antimicrobial effect of the prepared mixture, showing a large promoted antimicrobial reduction against *K. pneumoniae* and *C. albicans*, i.e., 82.60% and 85.60%, respectively. The antibacterial reduction of mixture of EP-PHB and PEG against *K. pneumoniae* (82.60%) is higher than that of individual EP-PHB (49.60%) and PEG (70.00%). Especially for the effect against *C. albicans*, the antimicrobial reduction of 85.60% was achieved by EP-PHB and PEG, which show no antifungi effect individually. This synergy had more significance than those that just promote the effect of originally antimicrobial agents [13,14,15,16,20]. Besides, the raw PHB powder has antibacterial reduction of only 60.28%, 0 and 82.40% against *S. aureus*, *K. pneumoniae* and *C. albicans*, respectively, whereas the mixture of PHB powder and PEG after stirring 4 h under 150 °C achieve obviously promoted antibacterial effect, i.e., 92.22%, 99.44% and 87.70% for *S. aureus*, *K. pneumoniae* and *C. albicans*, respectively (Table 2 and Figure 3). We further tested the antimicrobial effect of S-PHB (5 mg/mL) and S-PHB/PEG (10 mg/mL, 5 mg/mL for each). As seen in Table 2, compared with 10 mg/mL S-PHB, the antimicrobial effect of the lower concentration of S-PHB (5 mg/mL) was significantly reduced, especially *C. albicans*, whereas S-PHB (5 mg/mL) achieved much better sterilization effect after mixed with PEG (5 mg/mL). Therefore, these phenomena confirmed a synergistic antimicrobial effect between PHB oligomer and PEG. Additionally, it can be seen that the main influencing factors for antimicrobial effect, including concentration of PHB oligomer; degree of polymerization and temperature, i.e., high concentration; high temperature for reaction between PHB powder and PEG; and low degree of polymerization facilitated better antimicrobial property.

### 3.2. Investigation on Synergistic Mechanism between PHB Oligomer and PEG

After the discovery of synergy between PHB and PEG, three presumptions are raised as generation of new antimicrobial components, degradation of PHB macromolecules and dissolution/dispersion of PHB oligomer by PEG.

#### 3.2.1. Hypothesis of Generation of New Antimicrobial Components

**Figure 4:**
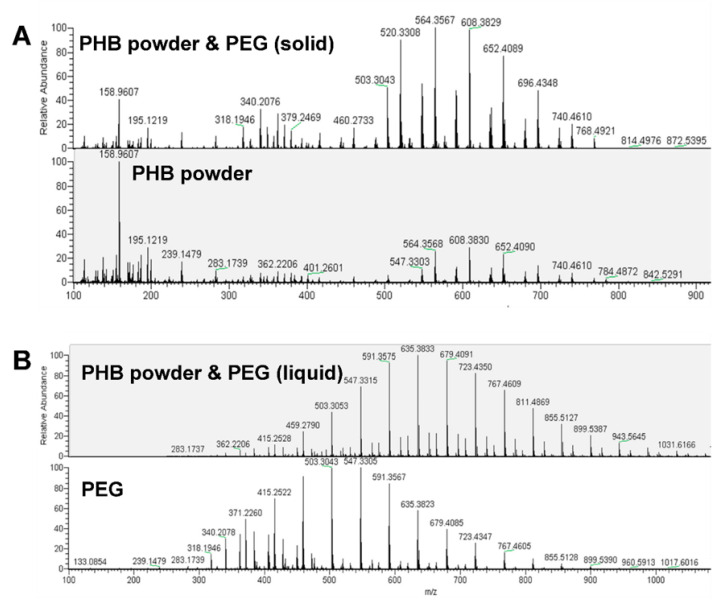
Mass spectrum of PHB powder and PEG after heat treatment. (**A**) Comparison between PHB power and the solid part of mixture of PHB powder and PEG after heat treatment. (**B**) Comparison between PEG and the liquid part of mixture of PHB powder and PEG after heat treatment.

The mixture of PHB powder and PEG after treatment under 150 °C possesses higher antibacterial reduction than each of the individual components. Some antimicrobial agents, such as reactive oxygen species [21], and radical [22] and new molecules [23], may be produced as a result of possible chemical reaction between the mixing components, leading to a better antimicrobial effect than each individual component. To see if this hypothesis was true or false, the mass spectrum (MS) was carried out for the solid and liquid part of mixture of PHB powder and PEG after heat treatment, as well as the individual PHB powder and PEG after heat treatment under 150 °C for 4 h. It can be seen from Figure 4A that there is no difference between PHB powder and the solid part of mixture of PHB powder and PEG after heat treatment, which means that no new solid antimicrobial component is produced after the reaction of PHB powder and PEG under high temperature. Similarly, the mass spectra of liquid part of mixture of PHB powder and PEG are compared with those of PEG in Figure 4B, showing no new chemical component is produced, whereas the average degree of polymerization of PEG increases approximately from 11 to 14 after heat treatment, and the corresponding number-average molecular weight increases from 540 to 673, illustrating the further polymerization of PEG rather than reaction with PHB power. Therefore, the hypothesis of generation of new antimicrobial components fails due to the constant chemical component observed by MS.

#### 3.2.2. Hypothesis of Degradation of PHB Macromolecules

**Figure 5:**
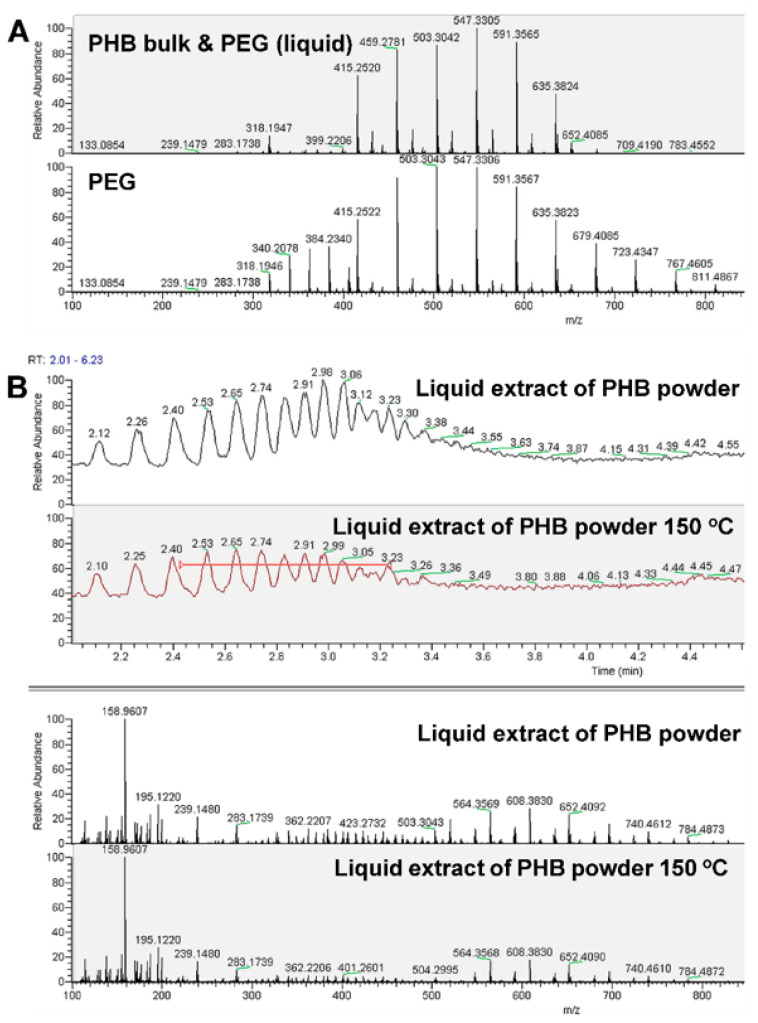
HPLC-MS analysis on hypothesis of degradation of PHB macromolecules. (**A**) Comparison between PEG and liquid part of mixture of PHB bulk and PEG after treatment under 150 °C for 4 h. (**B**) Extract of PHB powder before and after treatment under 150 °C for 4 h with solvent of methanol: dichloromethane = 1:1.

The second hypothesis is the degradation of PHB macromolecules, which can produce more PHB oligomer for better antimicrobial performance. Actually, it has been found previously that lower degree of polymerization facilitates better antimicrobial effect [18]. Therefore, PHB with higher degree of polymerization, i.e., PHB bulk, was extracted from the raw PHB powder, followed by reaction with PEG under 150 °C for 4 h, to see if the PHB macromolecules can be degraded by PEG under high temperature. The results are present in the mass spectrum in Figure 5A. It is supposed that PHB oligomer with low degree of polymerization should exist in the liquid part of mixture of PHB bulk and PEG after treatment, whereas there are no peaks of degraded PHB in the spectra showing almost the same peaks as the spectra of PEG, and a number-average of molecular weights of 524; thus, the hypothesis of degradation of PHB macromolecules is also not verified.

Actually, as the melting temperature of PHB is about 180 °C [24] and the thermal degradation temperature is over 190 °C [25], the heat treatment under 150 °C is insufficient for degrading the PHB macromolecules, even if it is in the powder state with high specific surficial area. Figure 5B exhibits the mass spectrum of liquid extract of raw PHB powder before and after heat treatment, showing that only PEG with low degree of polymerization exists, rather than the PHB oligomer through thermal degradation.

#### 3.2.3. Hypothesis of Dissolution/Dispersion of PHB Oligomer by PEG

PEG has very good hydrophilicity and is commonly used for enhancing hydrophilicity of functional materials through compounding [26,27] or grafting [28]. PHB oligomer is relatively hydrophobic material, especially for those with high degree of polymerization. The exist of PEG in raw PHB powder might facilitate the dissolution and dispersion of PHB oligomer therein into water, i.e., antimicrobial solution with bacteria. 

**Figure 6:**
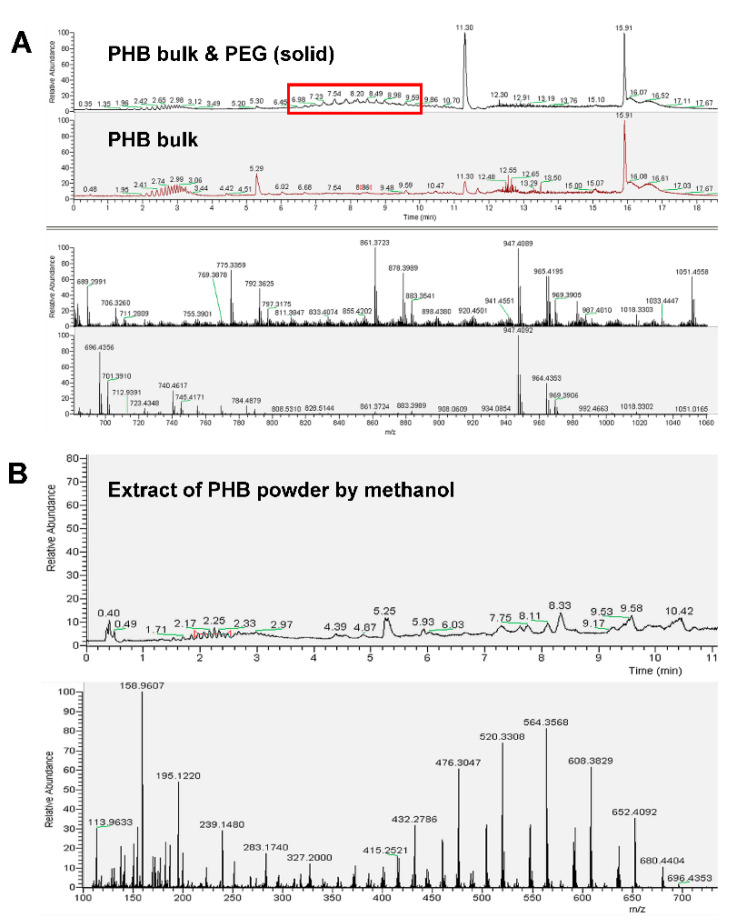
HPLC-MS analysis on hypothesis of dissolution/dispersion of PHB oligomer by PEG. (**A**) Comparison of PHB bulk and the solid part of mixture of PHB bulk and PEG after heat treatment under 150 °C for 4 h. (**B**) Extract of PHB powder with methanol.

**Figure 7:**
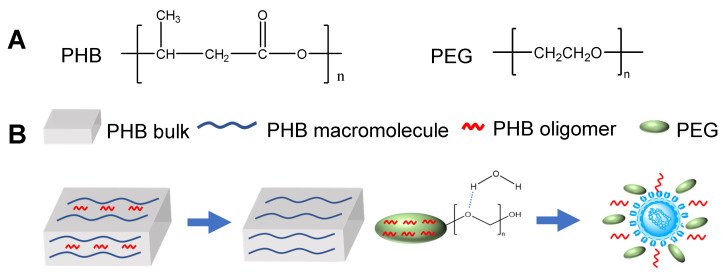
Schematic illustration of hypothesis of dissolution/dispersion of PHB oligomer by PEG. (**A**) Chemical structure of PHB and PEG. (**B**) Two steps are presented as dissolution/dispersion and synergistic antimicrobial effect.

For verifying this hypothesis, the PHB bulk (extracted from raw PHB powder with mainly macromolecules) was mixed with PEG (1:1) prior to a heat treatment under 150 °C for 4 h. The solid part after centrifugation was tested by HPLC-MS with solvent of methanol: dichloromethane = 1:1 (Figure 6A). Compared with the PHB bulk (same condition of treatment and test as above), new regular peaks appear during the eluting time between 6 min and 10 min, which are determined as PHB oligomer. Nine regular peaks of PHB oligomer appear, illustrating that about nine oligomers with different degrees of polymerization are included, and the polarity decreases with a higher degree of polymerization. This phenomenon illustrates that the PEG can promote the dissolution/dispersion of PHB oligomer in bulk into solvent/solution, thus confirming the hypothesis (Figure 7). Compared with the single function of PEG as formation of nanoparticle [29,30] or retention of water [31], PEG had double functions in this synergistic antimicrobial mechanism, i.e., promoting dissolution/dispersion, as well as working as an antimicrobial component. Though the E-PHB oligomer can be dissolved in methanol, PHB oligomer cannot directly be extracted from raw PHB powder by methanol (only tiny PEG exists in the extract, Figure 6B). Therefore, it is concluded that larger dose of PEG facilitates better affinity of PHB oligomer to solvent by dissolution/dispersion. The high hydrophilicity, low cost, biocompatible, eco-friendliness and cost-effectiveness of PEG assist its applications in biomedical devices (e.g., wound dressing), personal hygiene (e.g., antimicrobial hand wash cream) and food packaging (e.g., antimicrobial perseverative film).

## 4. Conclusions

A synergistic antimicrobial effect was discovered between PHB oligomer and PEG after comparison of a series of antimicrobial tests against gram-positive bacteria, gram-negative bacteria and fungi, followed by HPLC-MS analysis. Three proposed hypothetic mechanisms were tested by the experiments. The results show that there is neither new produced chemical component nor degradation/condensation of PHB after the compounding of PHB and PEG. The synergistic antimicrobial effect is due to the dissolution/dispersion of PHB oligomer by PEG, which has a good affinity to both the PHB and aqueous antimicrobial solution. This work reveals a new way to produce effective antimicrobial agent from bio-based PHB powder, reduce the minimal inhibitory concentration of pure PHB, and reduce the cost of production. It facilitates the cost-effective applications of bio-based PHB powder and synthesized PHB oligomer through improving the hydrophilicity.

## Figures and Tables

**Table 1:** Notions of materials, products and experiments.

PHB	Poly (3-hydroxybutyrate)
PHB powder	Raw material produced by fermentation
E-PHB	PHB oligomer extracted from PHB powder
EP-PHB	PHB oligomer extracted from PHB powder followed by further purification
S-PHB	PHB oligomer synthesized chemically by polymerization
PEG	Polyethylene glycol
EP-PHB and PEG	EP-PHB oligomer reacts with PEG (1:1, 150 °C, 4 h)
PHB powder and PEG	PHB powder reacts with PEG (1:1, room temperature, 4 h)
PEG heat treatment	Heat PEG at 150 °C for 4 h
PHB bulk	PHB polymer extracted from PHB powder, mainly containing macromolecules.
PHB bulk and PEG	PHB bulk reacts with PEG at 150 °C for 4 h
PHB powder heat treatment	Heat PHB powder at 150 °C for 4 h
Extract of PHB powder (methanol)	Extract of PHB powder by methanol dissolution, filtration and evaporation.

**Table 2:** Synergy between PHB oligomer and PEG.

Sample	Concentration mg/mL	Degree of Polymerization	Antimicrobial Property		
			*S. aureus*	*K. pneumoniae*	*C. albicans*
S-PHB oligomer [18]	10	≤6	>99.99%	>99.99%	>99.99%
S-PHB oligomer	5	≤6	>99.99%	91.60%	61.50%
S-PHB oligomer and PEG	10 (5 for each)	N.A.	>99.99%	98.30%	96.15%
E-PHB oligomer [18]	10	8~15	>99.99%	94.26%	91.95%
EP-PHB oligomer	10	8~15	>99.99%	49.60%	0
EP-PHB oligomer and PEG (1:1, 150 °C, 4 h)	10 (5 for each)	N.A.	>99.99%	82.60%	85.60%
PEG	10	≈14	0	70.00%	0
PHB powder	10	10,000~20,000	60.28%	0	82.40%
PHB powder and PEG (1:1, room temperature, 4 h)	10 (5 for each)	N.A.	67.22%	79.02%	38.50%
PHB powder and PEG (1:1, 150 °C, 4 h)	10 (5 for each)	N.A.	92.22%	99.44%	87.70%
